# The Interactions between ZnO Nanoparticles (NPs) and α-Linolenic Acid (LNA) Complexed to BSA Did Not Influence the Toxicity of ZnO NPs on HepG2 Cells

**DOI:** 10.3390/nano7040091

**Published:** 2017-04-24

**Authors:** Yiwei Zhou, Xin Fang, Yu Gong, Aiping Xiao, Yixi Xie, Liangliang Liu, Yi Cao

**Affiliations:** 1 Institute of Bast Fiber Crops, Chinese Academy of Agricultural Sciences, Changsha 410205, China; zyw97@hotmail.com (Y.Z.); lastchance39@163.com (X.F.); aipingxiao@yahoo.com (A.X.); 2 Key Laboratory of Environment-Friendly Chemistry and Applications of Ministry Education, Laboratory of Biochemistry, College of Chemistry, Xiangtan University, Xiangtan 411105, China; gongyu910803@sina.com (Y.G.); xieyixige@xtu.edu.cn (Y.X.)

**Keywords:** HepG2 cells, α-linolenic acid (LNA), ZnO nanoparticles, interaction

## Abstract

Background: Nanoparticles (NPs) entering the biological environment could interact with biomolecules, but little is known about the interaction between unsaturated fatty acids (UFA) and NPs. Methods: This study used α-linolenic acid (LNA) complexed to bovine serum albumin (BSA) for UFA and HepG2 cells for hepatocytes. The interactions between BSA or LNA and ZnO NPs were studied. Results: The presence of BSA or LNA affected the hydrodynamic size, zeta potential, UV-Vis, fluorescence, and synchronous fluorescence spectra of ZnO NPs, which indicated an interaction between BSA or LNA and NPs. Exposure to ZnO NPs with the presence of BSA significantly induced the damage to mitochondria and lysosomes in HepG2 cells, associated with an increase of intracellular Zn ions, but not intracellular superoxide. Paradoxically, the release of inflammatory cytokine interleukin-6 (IL-6) was decreased, which indicated the anti-inflammatory effects of ZnO NPs when BSA was present. The presence of LNA did not significantly affect all of these endpoints in HepG2 cells exposed to ZnO NPs and BSA. Conclusions: the results from the present study indicated that BSA-complexed LNA might modestly interact with ZnO NPs, but did not significantly affect ZnO NPs and BSA-induced biological effects in HepG2 cells.

## 1. Introduction

When nanoparticles (NPs) enter the biological environment, the (bio)molecules may be absorbed onto the surface of the NPs, which gives the NPs a biological identity different from the synthetic identity which consequently affects the biological effects of NPs [1]. One of the well-studied examples is the interaction between NPs and proteins, e.g., bovine serum albumin (BSA). Extensive studies have shown that the formation of protein corona onto the surface of NPs could alter the size, aggregation state, and interfacial properties of NPs, as well as the interactions between NPs and cells/tissues, which consequently changes the toxicity of NPs compared with the naked NPs [1,2,3]. In addition to proteins, recent studies also showed that the presence of nutrients could also define the toxicological responses of NPs [1,2]. For example, it has been shown that simultaneous exposure of rats to TiO_2_ NPs and glucose induced larger toxicological responses compared with the exposure to TiO_2_ NPs alone due to the effects of excessive glucose and/or the interactions between glucose and NPs [4]. Similarly, another study showed that the interactions between SiO_2_ NPs and food components, namely glucose and albumin, facilitated the uptake of NPs following oral exposure in rats. The authors suggested that it may be necessary to consider the interactions between SiO_2_ NPs and glucose for the risk assessment of SiO_2_ NPs via oral exposure [5]. The presence of vitamin C was shown to enhance the toxicity of ZnO NPs both in vivo and in vitro due to accelerated dissolution of ZnO, as well as enhanced uptake of Zn ions in the presence of vitamin C [6]. In contrast, flavonoids, such as quercetin and kaempferol, protected Caco-2 cells from exposure to Ag NPs due to the anti-oxidant properties of flavonoids [7,8]. Based on available reports, we have recently proposed that interactions between nutrients and NPs should be considered when evaluating the toxicity of NPs [9].

Recently it was shown that the interactions between lipids and NPs may affect the toxicity of NPs and, thus, needs further study. For example, the presence of a typical saturated fatty acid (SFA), palmitic acid (100 µM) promoted multi-walled carbon nanotube-mediated adhesion of THP-1 monocytes onto human endothelial cells without an effect on production of the intracellular reactive oxygen species (ROS) or depletion of glutathione (GSH) [10]. In contrast, co-exposure to 200 µM palmitate and ZnO NPs did not significantly affect THP-1 monocyte adhesion, but induced higher cytotoxicity to human endothelial cells due to combined toxicity of palmitate and ZnO NPs [11]. In Caco-2 cells, the presence of 500 µM palmitic acid enhanced the cytotoxicity of ZnO NPs associated with increased mitochondrial ROS [12]. At much lower concentrations, 50 µM, but not 10 µM, palmitate enhanced the toxicity of ZnO NPs to lysosomes, but not NP-induced accumulation of intracellular Zn ions in THP-1 macrophages [13]. All of these studies, in combination, indicated that the presence of SFA might promote the adverse effects of NPs due to the intrinsic toxicity of SFA. However, little is known about the interactions between unsaturated fatty acids (UFA) and NPs, as well as the influence on the toxicity of NPs.

In the present study, α-linolenic acid (LNA) was used as a model for UFA and its interactions with ZnO NPs on the toxicity of NPs on human liver HepG2 cells were studied. LNA was selected because it is an essential fatty acid with 18 carbons and three cis double bonds. LNA has been reported to have beneficial effects against certain inflammatory, cardiovascular, and neurodegenerative diseases [14,15,16,17,18]. Moreover, LNA has not been reported to induce serious health problems, which indicates that it is safe as a dietary ingredient [19]. ZnO NPs were studied because they are important NPs with many biomedical applications, such as anti-tumor, anti-bacterial, and anti-diabetic functions [20,21,22,23]. Indeed, ZnO NPs are among one of the most popular metal-based NPs used in commercially-available products, which may result in NP exposure via dermal, inhalational, or oral contact [24]. Thus, it is expected that human beings are exposed to ZnO NPs in modern society and it is necessary to assess the toxicity of ZnO NPs to cells associated with potential targets. HepG2 cells were selected as an in vitro model for hepatocytes because liver cells are among one of the potential targets following ZnO NP exposure, and animal studies showed that ZnO NPs via different exposure routes may lead to the accumulation of Zn in the liver [25,26,27]. Moreover, it is well-known that the liver is a main site for the metabolism of lipids. As such, it is possible that the interactions between ZnO NPs and lipids will likely happen in the liver. To indicate the interactions between ZnO NPs and LNA the changes of hydrodynamic sizes, zeta potential, UV-Vis, and fluorescence and synchronous fluorescence spectra were investigated. To indicate the possible effects of interactions between ZnO NPs and LNA on the toxicity of NPs, cytotoxicity, intracellular superoxide, accumulation of Zn ions, and the release of inflammatory cytokines in HepG2 cells exposed to ZnO NPs with or without the presence of LNA were studied.

## 2. Results

### 2.1. Characteristics of ZnO NPs

**Figure 1:**
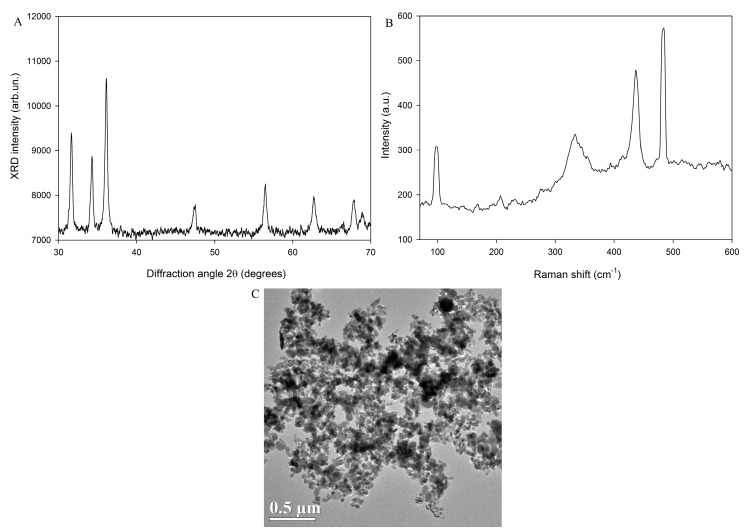
Characteristics of ZnO NPs (code: XFI06). (**A**) the XRD spectrum of ZnO NPs. (**B**) the Raman spectrum of ZnO NPs. (**C**) TEM picture of ZnO NPs.

The X-ray diffractograms (XRD) and Raman spectrum of ZnO NPs (code: XFI06) are shown in Figure 1A,B, which indicated the presence of ZnO NPs without impurities. The corresponding XRD pattern (Figure 1A) indicated the hexagonal phase of NPs. The XRD size was calculated as 22.3 nm. The Brunauer Emmett Teller (BET) surface area was measured as 19.072 m^2^/g. Figure 1C shows the spheroidal or nearly spheroidal morphology of ZnO NPs detected by transmission electron microscopy (TEM) analysis. The primary size of ZnO NPs was measured as 32.5 ± 9.8 nm based on the measurement of 50 randomly-selected NPs. The hydrodynamic size of freshly-prepared ZnO NPs was measured as 234.4 ± 3.5 nm, which indicated the presence of aggregates and/or agglomerates of NPs in suspension. The zeta potential was measured as −15.5 ± 0.3 mV.

### 2.2. The Interactions between ZnO NPs and LNA

#### 2.2.1. Changes of Hydrodynamic Size and Zeta Potential

**Figure 2:**
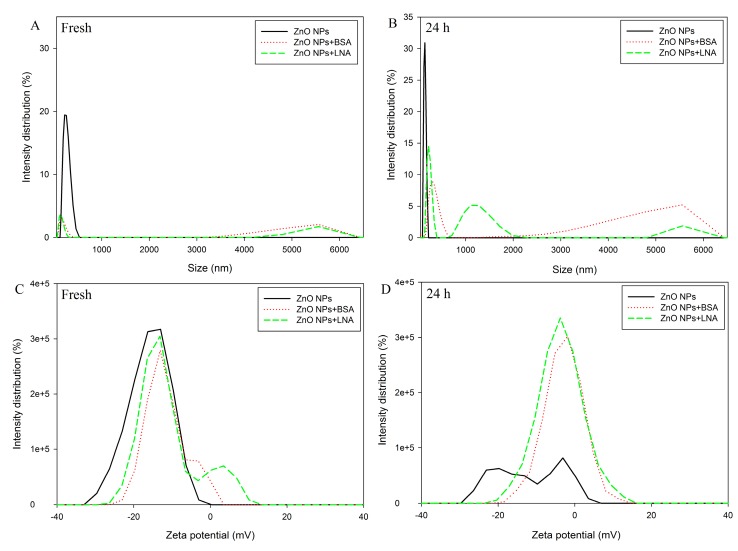
The hydrodynamic size (**A**,**B**) and zeta potential (**C**,**D**) of freshly prepared (**A**,**C**) and aged for 24 h (**B**,**D**) ZnO NPs (code: XFI06) suspended in distilled and deionized water (DDW), 0.5% BSA, or 200 µM α-linolenic acid (LNA; complexed to BSA; referred as LNA). The sizes smaller than 50 nm are not shown in this figure.

The changes of hydrodynamic size and zeta potential of freshly-prepared and aged ZnO NPs suspended in different solutions are shown in Figure 2. Since LNA, as a fatty acid, is not soluble in water, BSA was used to complex LNA [12]. As such, an equal amount of BSA was added in control groups to compare with the effects of LNA for the rest of the experiments. BSA-complexed LNA is referred to as LNA for simplicity throughout the manuscript. BSA or LNA showed hydrodynamic sizes lower than 10 nm; therefore, hydrodynamic sizes lower than 10 nm were not shown in Figure 2. When BSA or LNA was present, there was an apparent increase of the hydrodynamic sizes of both freshly-prepared (Figure 2A) and aged ZnO NP suspension (Figure 2B). For the zeta potential (Figure 2C,D), the aged samples of ZnO NPs showed little to no zeta potential distribution, which was not observed when BSA or LNA was present.

#### 2.2.2. Changes of UV-Vis, Fluorescence, and Synchronous Fluorescence Spectra

**Figure 3:**
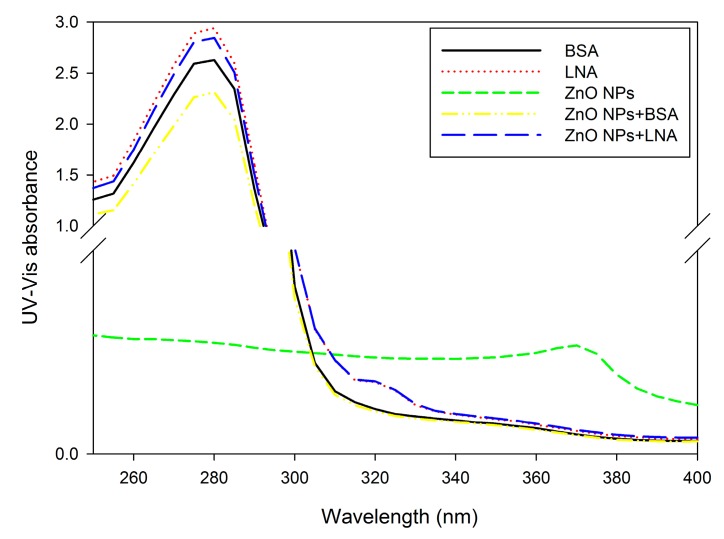
The UV-Vis spectra of bovine serum albumin (BSA), α-linolenic acid (LNA complexed to BSA; referred as LNA) and ZnO NPs (code: XFI06) suspended in distilled and deionized water (DDW), 0.5% BSA, or 200 µM LNA. Distilled and deionized water (DDW) was used as a blank.

The UV-Vis spectra of different suspensions are shown in Figure 3. ZnO NPs showed a peak of absorbance at 360–370 nm, which disappeared when BSA or LNA was present. BSA or LNA showed a peak of absorbance at about 280 nm. The peak was not shifted, but the absorbance was lowered by the presence of 16 µg/mL ZnO NPs.

**Figure 4:**
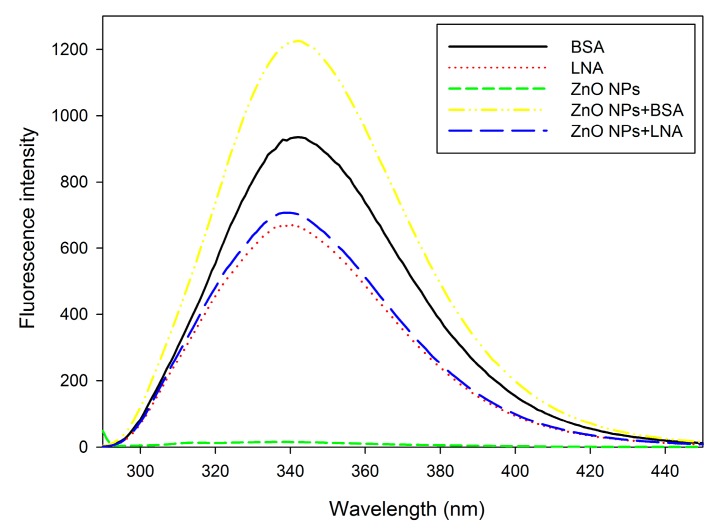
The fluorescence spectra (λ = 280 nm) of bovine serum albumin (BSA), α-linolenic acid (LNA; complexed to BSA; referred as LNA) and ZnO NPs (code: XFI06) suspended in distilled and deionized water (DDW), 0.5% BSA, or 200 µM LNA.

The fluorescence spectra at λ = 280 nm are shown in Figure 4. The peak emission band of BSA or LNA is about 340 nm. The peak was not shifted, but the emission of fluorescence was increased by the presence of 16 µg/mL ZnO NPs.

**Figure 5:**
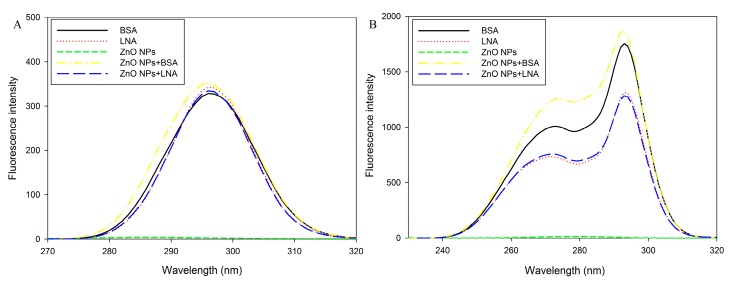
Synchronous fluorescence spectra of bovine serum albumin (BSA), α-linolenic acid (LNA; complexed to BSA; referred as LNA) and ZnO NPs (code: XFI06) suspended in distilled and deionized water (DDW), 0.5% BSA, or 200 µM LNA. (**A**) Δλ = 15 nm; (**B**) Δλ = 60 nm.

The synchronous fluorescence spectra at Δλ = 15 nm and Δλ = 60 nm are shown in Figure 5A,B, respectively. The peaks of fluorescence emission of BSA or LNA at Δλ = 15 nm and Δλ = 60 nm are at about 295 nm for both cases and, again, the peaks were not obviously shifted by the presence of 16 µg/mL ZnO NPs. However, the fluorescence emission of BSA was increased by the presence of ZnO NPs, whereas the fluorescence emission of LNA was slightly decreased by the presence of ZnO NPs.

### 2.3. Cytotoxicity

#### 2.3.1. Cellular Viability (Cell Counting Kit-8, CCK-8 Assay)

**Figure 6:**
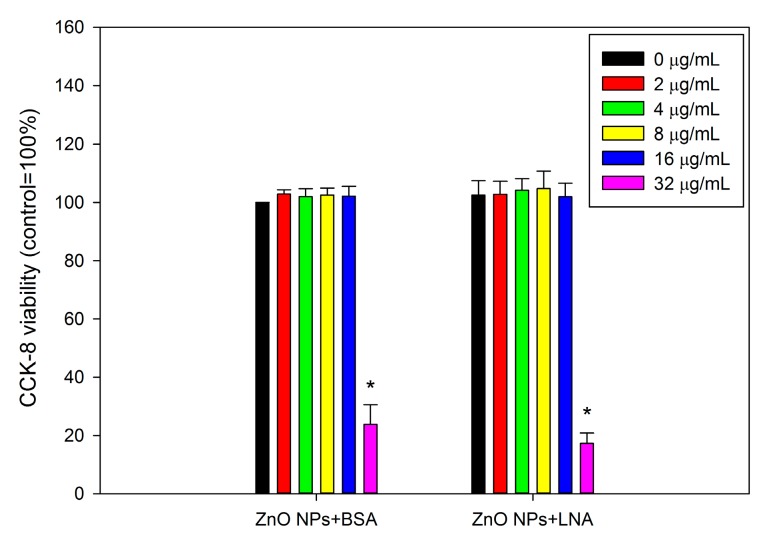
Cellular viability of HepG2 cells as assessed by cell counting kit-8 (CCK-8) assay. The cells were exposed to various concentrations of ZnO NPs (code: XFI06) with the presence of bovine serum albumin (BSA) or 200 µM α-linolenic acid (LNA; complexed to BSA; referred to as LNA) for 24 h, and the cellular viability was measured by CCK-8 assay. *, *p* < 0.01, compared with control, ANOVA.

Since LNA was complexed to BSA to increase the solubility, BSA was added to control groups and the effects of ZnO NPs in the presence of LNA were compared with the effects of ZnO NPs in the presence of BSA for the rest of the experiments. The cytotoxicity of ZnO NPs to HepG2 cells is shown in Figure 6. ANOVA indicates a single factor effect of concentrations of NPs (*p* < 0.01), whereas the presence of LNA did not significantly affect cellular viability (*p* > 0.05). Exposure to 32 µg/mL ZnO NPs with the presence of BSA or LNA was associated with significantly increased cytotoxicity in HepG2 cells (*p* < 0.01).

#### 2.3.2. Lysosomal Damage (Neutral Red Uptake and Acridine Orange Assay)

**Figure 7:**
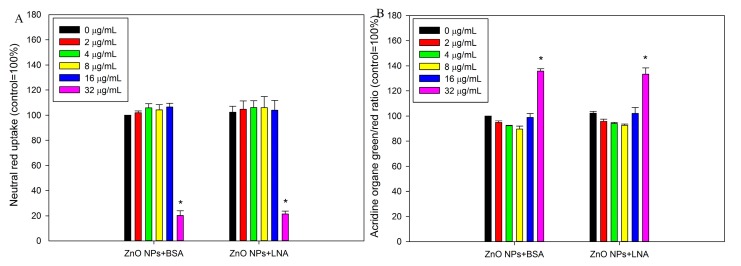
The damage of lysosomes as assessed by neutral red uptake assay (**A**) or acridine orange staining (**B**). HepG2 cells were exposed to various concentrations of ZnO NPs (code: XFI06) with the presence of bovine serum albumin (BSA) or α-linolenic acid (LNA; complexed to BSA; referred to as LNA) for 24 h (**A**) or 3 h (**B**), and neutral red uptake assay (**A**) or acridine orange staining (**B**) were used to indicate the integrity of lysosomes. *, *p* < 0.01, ANOVA, compared with control.

Neutral red uptake and acridine orange assay were further used to indicate the damage to lysosomes and the results are shown in Figure 7. Both neutral red uptake (Figure 7A) and acridine orange assay (Figure 7B) indicated that exposure to 32 µg/mL ZnO NPs with the presence of BSA or LNA was associated with significantly increased damage to lysosomes (*p* < 0.01). The presence of LNA did not significantly increase lysosomal damage, and ANOVA indicates no interaction between concentrations of ZnO NPs and LNA (*p* > 0.05).

### 2.4. Intracellular Superoxide

**Figure 8:**
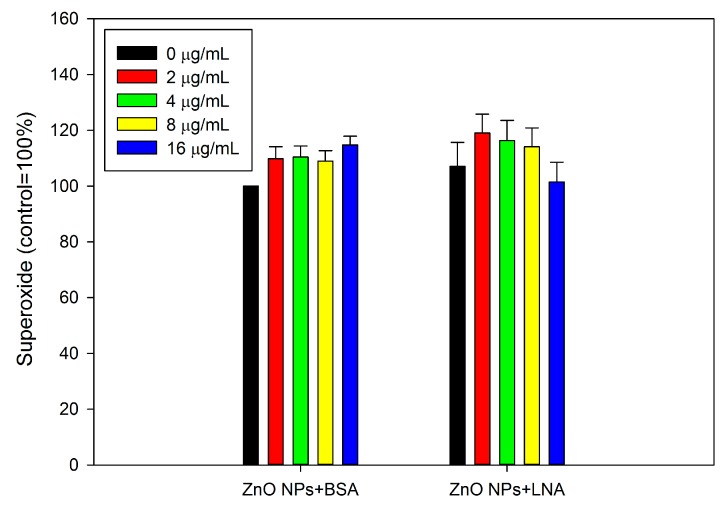
The intracellular superoxide. HepG2 cells were exposed to various concentrations of ZnO NPs (code: XFI06) with the presence of bovine serum albumin (BSA) or α-linolenic acid (LNA complexed to BSA; referred to as LNA) for 3 h, and the intracellular superoxide was measured by using dihydroethidium (DHE).

Figure 8 shows the production of intracellular superoxide in HepG2 cells. Exposure to non-cytotoxic concentrations of ZnO NPs with the presence of BSA or LNA did not significantly affect intracellular superoxide concentrations (*p* > 0.05). The presence of LNA did not significantly induce intracellular superoxide concentrations, and ANOVA indicates no interaction between concentrations of ZnO NPs and the presence of LNA (*p* > 0.05).

### 2.5. Accumulation of Intracellular Zn Ions

**Figure 9:**
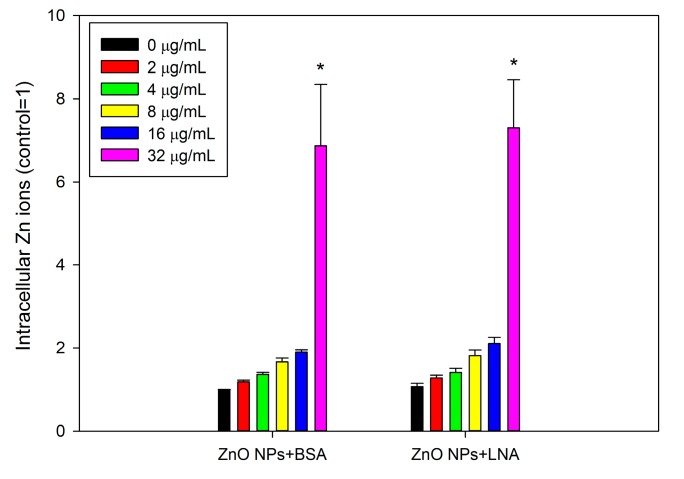
The accumulation of intracellular Zn ions. HepG2 cells were exposed to various concentrations of ZnO NPs (code: XFI06) with the presence of bovine serum albumin (BSA) or α-linolenic acid (LNA complexed to BSA; referred to as LNA) for 3 h, and the accumulation of intracellular Zn ions was measured by using a fluorescent probe. *, *p* < 0.01, ANOVA, compared with control.

As shown in Figure 9, exposure to ZnO NPs dose-dependently promoted the accumulation of intracellular Zn ions in HepG2 cells, and there was a statistically significant increase of intracellular Zn ions after exposure to 32 μg/mL ZnO NPs with the presence of BSA or LNA (*p* < 0.01). However, the presence of LNA did not significantly affect the accumulation of intracellular Zn ions, and ANOVA indicates no interaction between concentrations of ZnO NPs and LNA (*p* > 0.05).

### 2.6. Release of Inflammatory Cytokines

**Figure 10:**
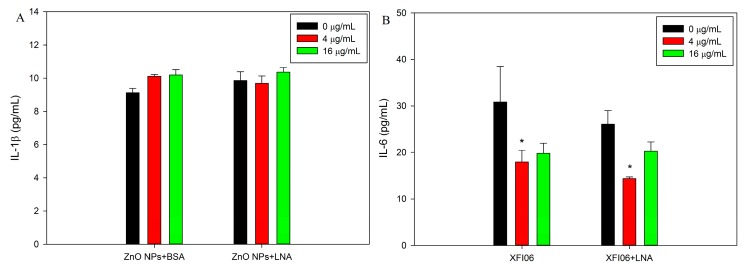
The release of inflammatory cytokines interleukin-1β (IL-1β; (**A**)) or IL-6 (**B**). HepG2 cells were exposed to various concentrations of ZnO NPs (code: XFI06) with the presence of bovine serum albumin (BSA) or α-linolenic acid (LNA; complexed to BSA; referred to as LNA) for 24 h, and the release of IL-1β and IL-6 was measured by ELISA. *, *p* < 0.05, ANOVA, compared with control.

Figure 10 depicts the release of inflammatory cytokines, namely IL-1β and IL-6, in HepG2 cells. The concentrations of IL-1β were low in all the samples and did not significantly change after exposure to ZnO NPs with the presence of BSA or LNA (*p* > 0.05, Figure 10A). In contrast, exposure to ZnO NPs dose-dependently decreased the release of IL-6, and a statistically significant decrease of IL-6 was observed after exposure to 4 µg/mL ZnO NPs with the presence of BSA or LNA (*p* < 0.05, Figure 10B). The presence of LNA did not significantly affect the release of cytokines, and ANOVA indicates no interaction between concentrations of ZnO NPs and LNA on the release of cytokines (*p* > 0.05).

## 3. Discussion

In the present study, the interactions between ZnO NPs and BSA or LNA (complexed to BSA to increase the solubility) were indicated by the changes of hydrodynamic size, zeta potential, UV-Vis, and fluorescence and synchronous fluorescence spectra. As expected, the presence of BSA or LNA complexed to BSA increased the hydrodynamic sizes both of freshly-prepared and aged NPs (24 h), and the zeta potential of ZnO NPs was maintained after aged for 24 h (Figure 2). We did not measure further the hydrodynamic sizes of ZnO NPs in cell culture medium due to the interference of components present in the medium. However, it is expected that BSA or LNA will show similar effects on the hydrodynamic sizes and Zeta potential of ZnO NPs when added into the cell culture medium. Previously, it was shown that the salt of SFA or SFA complexed to BSA may coat NPs, showing as altered hydrodynamic sizes and/or zeta potential of NPs [10,11,12,13]. The results from the present study further indicated that the presence of BSA or LNA complexed to BSA could coat ZnO NPs and, consequently, stabilize the NPs.

The UV-Vis, fluorescence, and synchronous fluorescence spectra of different suspensions were recorded to provide more information about the interactions between BSA/LNA complexed to BSA and ZnO NPs. For the UV-Vis spectra, the ZnO NPs showed a peak of absorbance at 360–370 nm (Figure 3), which has been observed before, as we previously reported [13]. However, unlike the binding of palmitate to ZnO NPs, which increased the absorbance [13], the absorbance of ZnO NPs at 360–370 nm disappeared with the presence of BSA or LNA complexed to BSA. A recent study also showed the disappearance of this band with the covalent coating of BSA to ZnO NPs, which has been suggested to be due to the different refractiveness of the microenvironment of NPs and/or scattering, as compared to the non-covalently coated NPs [28].

For all of the assays, BSA or LNA complexed to BSA exhibited characteristic bands of absorption or fluorescence emission, and the presence of ZnO NPs did not obviously shift the band but altered the intensity of the band (Figure 3, Figure 4 and Figure 5). It has been shown before that the covalent binding of BSA to ZnO NPs could lead to the shift of the band of absorption and fluorescence emission, which indicated the changes of hydrophobicity of tyrosine and tryptophan residues of BSA [28,29]. Moreover, the Raman spectrum further confirmed that the structures in BSA, namely α-helix, β-sheet, and unstructured folding, were changed by the presence of ZnO NPs [29]. However, we noticed that previous studies used relatively high concentrations of ZnO NPs (e.g., 50 µg/mL) [28,29], whereas here only 16 μg/mL ZnO NPs was used. This concentration of ZnO NPs was also among the concentrations used for HepG2 cell exposure. At the concentration used in this study, the presence of ZnO NPs did not shift, but changed the intensity of the band of absorption or fluorescence emission. These results, in combination, indicated that the binding of ZnO NPs to BSA or LNA complexed to BSA did not result in changes of the conformation of proteins. Compared with the changes of UV-Vis and fluorescence spectra of BSA induced by ZnO NPs, the presence of ZnO NPs only slightly changed the intensity of UV-Vis and fluorescence spectra of LNA (Figure 3, Figure 4 and Figure 5), which indicated that the interactions between ZnO NPs and LNA could be modest without conformational changes of proteins.

The impact of interactions between LNA (complexed to BSA) and ZnO NPs on the toxicity of NPs to HepG2 cells were then studied, in comparison with ZnO NPs in the presence of BSA. ZnO NPs in the presence of BSA induced cytotoxicity to HepG2 cells as increased damage to mitochondria and lysosomes (Figure 6 and Figure 7). This is consistent with a number of previous reports, which showed that ZnO NPs could induce cytotoxicity on both normal and cancerous liver cells [30,31,32,33]. Exposure to 200 µM LNA did not significantly affect the cytotoxicity of HepG2 cells. Previously, it was shown that exposure to plant oils rich in LNA did not significantly affect the viability of fibroblasts in vitro [34], and uptake of LNA in animals or human beings did not induce adverse health effects [19]. Our results in combination with previous reports indicated low toxic potential of LNA. With the presence of LNA, the cytotoxicity of ZnO NPs to HepG2 cells was not significantly affected. To the best of our knowledge, there is no previous report about the effect of UFA on the toxicity of ZnO NPs. However, it has been shown before that the mixture of oleic acid (a typical UFA) and palmitic acid did not significantly affect the cytotoxicity of ZnO NPs to Caco-2 cells, whereas palmitic acid alone significantly promoted the cytotoxicity of ZnO NPs [12].

ZnO NPs are partially soluble, and it has been strongly accepted that the dissolution of ZnO NPs inside and/or outside the cells and, subsequently, the release of Zn ions, is responsible for the toxicity of ZnO NPs [20,35]. To address this issue, the accumulation of intracellular Zn ions was quantified by using a fluorescence probe. Due to the severe cytotoxicity and detachment of cells after 24 h exposure to high concentrations of ZnO NPs (with the presence of BSA or LNA), the cells were exposed for 3 h for the quantification of Zn accumulation, as well as other assays, using fluorescence probe. In our recent studies, we showed that it is possible to measure the endpoints by fluorescence probes when the exposure time was shortened as 3 h [11,13]. Moreover, we have also shown that ZnO NP induced accumulation of Zn ions and ROS production in THP-1 macrophages were similar after 3 h and 24 h exposure [13,36]. This indicated that these endpoints in response to ZnO NPs may already reach a maximum after 3 h exposure. The results from this study showed significantly increased intracellular Zn ions after exposure to ZnO NPs and BSA at cytotoxic concentrations (Figure 9), whereas the generation of superoxide was not significantly affected (Figure 8). Thus, it is possible that the accumulation of intracellular Zn ions is responsible for the toxicity of ZnO NPs [35]. With the presence of LNA, ZnO NPs and BSA-induced Zn ion accumulation was not significantly affected in HepG2 cells. Previous work showed that the presence of vitamin C enhanced the toxicity of ZnO NPs due to the enhanced accumulation of intracellular Zn ions [6], whereas surface modifications of ZnO NPs could reduce the cytotoxicity of NPs by the reduction of NP uptake and, subsequently, the accumulation of intracellular Zn ions [37]. Thus, the unaltered cytotoxicity of ZnO NPs and BSA to HepG2 cells with the presence of LNA could be explained in that LNA did not significantly promote the accumulation of Zn ions into the cells. This is also consistent with the modest interactions between ZnO NPs and LNA as observed by UV-Vis and fluorescence spectra (Figure 3, Figure 4 and Figure 5).

Exposure to NPs, including ZnO NPs, has been suggested to modulate inflammatory responses that are associated with oxidative stress [38,39]. Herein, the oxidative stress was indicated by the measurement of superoxide, whereas the inflammatory response was indicated by the measurement of the release of cytokines IL-1β and IL-6. Paradoxically, the release of IL-6 was significantly decreased after exposure to ZnO NPs in the presence of BSA (Figure 10), which indicated the anti-inflammatory potential. Meanwhile, superoxide was not significantly induced after exposure to non-cytotoxic concentrations of ZnO NPs in the presence of BSA (Figure 8). A previous study showed that tetrapod-like ZnO NPs inhibited the productions and mRNA expressions of inflammatory cytokines in human mast cells [40]. In another study, ZnO NPs were shown to inhibit inflammatory responses in interferon-γ plus lipopolysaccharide-stimulated macrophages [41]. Similarly, our recent study also showed that ZnO NPs reduced the release of inflammatory mediators from lipopolysaccharide-stimulated human endothelial cells [11]. These studies suggested that ZnO NPs may inhibit inflammation under certain conditions. Nevertheless, the results of the present study showed that superoxide and the release of inflammatory markers by ZnO NPs and BSA exposure were not significantly affected by the presence of LNA, which suggested that the interactions between LNA and ZnO NPs did not affect the toxicity of ZnO NPs on HepG2 cells.

## 4. Materials and Methods

### 4.1. Cell Culture 

The human liver cell line HepG2 (ATCC) was cultured in DMEM (Dulbecco’s Modified Eagle Medium)/high glucose medium (Hyclone, GE Healthcare) supplemented with 10% fetal bovine serum (Gibco, Australia) and 1% penicillin-streptomycin solution (Beyotime, Nantong, China) at 37 °C in a cell incubator. The cell culture medium was changed every 2–3 days, and HepG2 cells were sub-cultured at a ratio of 1:3 to 1:5 after trypsinization when near confluence. During the experimental period, the HepG2 cells were used within two months and 10 passages to keep their best characteristics. For all of the experiments, the cells were seeded at a density of 6 × 10^4^/well (24-well plates) or 1.5 × 10^4^/well (96-well plates) and grown for two days before exposure.

### 4.2. Preparation of LNA-BSA Solution

Since LNA, as a free fatty acid, is not water soluble, BSA was used to complex LNA as previously described [12]. Briefly, a stock solution of 200 mM LNA (purchased from Sigma-Aldrich, Saint Louis, MO, USA) was prepared in 96% EtOH and then diluted into 4 mM LNA in 10% BSA (Sigma-Aldrich)/Hanks’ solution (Beyotime, Nantong, China). The mixture was incubated at 37 °C, vortexing for about 30 min. Then, this solution was diluted in complete cell culture medium as 200 µM LNA (complexed to 0.5% BSA) for exposure. The complete cell culture medium containing 0.5% BSA and 0.1% EtOH was used for the control groups.

### 4.3. Particle Characteristics and Preparation

ZnO NPs (code XFI06; 20 nm) were purchased from Nanjing XFNANO Materials Tech Co., Ltd. (Nanjing, China) and thoroughly characterized by using XRD, Raman spectroscopy, TEM, BET surface area, and dynamic light scattering (DLS) in this study. XRD was used to determine the phase compositions and average crystallite sizes by a Bruker D8 Advanced diffractometer, and Raman spectra were recorded by using an inVia confocal Raman microscope (Renishaw, New Mills, Gloucestershire, UK). The morphology and structure of ZnO NPs were investigated by using TEM (FEI Tecnai G20, Hillsboro, OR, USA) accelerated at 200 kV. The primary size of ZnO NPs was estimated by using ImageJ software (NIH) based on the measurement of 50 randomly-selected NPs. The surface area was measured by using TriStarII 3020 (Micromeritics Corporate, Norcross, GA, USA). To expose the cells, a stock solution of 1.28 mg/mL particles in distilled and deionized water (DDW) containing 2% FBS was prepared by sonication continuously for two times of 8 min with continuously cooling on ice using an ultrasonic processor FS-250N (20% amplitude; Shanghai, China), and then diluted in complete cell culture medium. To indicate the stability of different ZnO NP suspensions, the hydrodynamic size distribution and zeta potential of 16 µg/mL ZnO NP suspended in DDW, 0.5% BSA or 200 µM LNA were measured after being freshly prepared or stored in a cell incubator for 24 h by using Zetasizer nano ZS90 (Malvern, Amesbury, UK).

### 4.4. UV-Vis Spectra of XFI06 in Different Suspensions

The UV-Vis spectra of 0.5% BSA, 200 µM LNA, 16 µg/mL ZnO NPs, 16 µg/mL ZnO NPs + BSA, and 16 µg/mL ZnO NPs + LNA were recorded by an Agilent Cary 60 UV-Vis spectrophotometer (Agilent Technologies, Santa Clara, CA, USA), and distilled and deionized water (DDW) was used as a blank.

### 4.5. The Fluorescence and Synchronous Fluorescence Spectra of XFI06 in Different Suspensions

The fluorescence and synchronous fluorescence spectra of 0.5% BSA, 200 µM LNA, 16 µg/mL ZnO NPs, 16 µg/mL ZnO NPs + BSA and 16 µg/mL ZnO NPs + LNA were recorded by using a Hitachi F4600 fluorescence spectrophotometer (Hitachi, Japan). For fluorescence spectra, the excitation wavelength was set as 280 nm. For synchronous fluorescence spectra, the initial excitation wavelength was set at 200 nm and up to 500 nm, and Δλ was set at 15 nm and 60 nm for tyrosine and tryptophan residues, respectively.

### 4.6. Cytotoxicity (CCK-8 Assay)

CCK-8 (cell counting kit-8) assay was used to assess the cytotoxicity induced by increasing concentrations of ZnO NPs on HepG2 cells with the presence of BSA or LNA. WST-8 (water-soluble tetrazolium-8) in CCK-8 reagent has been used as a substitute for the commonly-used MTT assay, and we have recently shown that both CCK-8 and MTT assays could obtain similar results, but CCK-8 assay was easier and faster [42]. CCK-8 is converted to water-soluble yellow formazan by mitochondria in living cells, therefore, CCK-8 could be used to reflect the cellular viability. The assay was done according to the manufacturer’s instructions (Beyotime, Nantong, China). Briefly, HepG2 cells on 24-well plates were exposed to 0 µg/mL, 2 µg/mL, 4 µg/mL, 8 µg/mL, 16 µg/mL, and 32 µg/mL ZnO NPs with the presence of BSA or 200 µM LNA for 24 h. After exposure, the cells were rinsed once with Hanks’ solution, and then incubated with cell culture medium containing 10% CCK-8 for 1 h. The yellow product was read at 450 nm with 690 nm as a reference by an ELISA reader (Synergy HT, BioTek, Woburn, MA, USA).

### 4.7. Neutral Red Uptake Assay and Acridine Orange Staining

To further indicate the damage to lysosomes, neutral red uptake assay and acridine orange staining were used. Neutral red is a dye that can accumulate in intact lysosomes of living cells and, thus, could be used to indicate the integrity of lysosomes. The assay was done by using a commercial kit according to the manufacturer’s instructions (Beyotime, Nantong, China). Briefly, HepG2 cells on 24-well plates were exposed for 24 h, as indicated above, rinsed, and then incubated with cell culture medium containing 5% neutral red. After staining for 2 h, the cells were rinsed once by using Hanks’ solution, and the neutral red incorporated into lysosomes was dissolved in lysis buffer provided by the kit. The red product was then read at 540 nm with 690 nm as a reference by an ELISA reader.

The increase of the acridine orange green/red fluorescence ratio could reflect the damage of lysosomes because it changes the fluorescence from red to green when it translocates from the damaged lysosomes into cytosol [13]. The assay was done as we previously described [13]. Briefly, after 3 h of exposure to various concentrations of ZnO NPs with the presence of BSA or 200 µM LNA, HepG2 cells on black 96-well plates were rinsed, stained with 5 µM acridine orange for 15 min, and then rinsed again to remove free probes. The green fluorescence was read at *E*_x_ 485 ± 20 nm and *E*_m_ 528 ± 20 nm, and the red fluorescence was read at *E*_x_ 530 ± 25 nm and *E*_m_ 645 ± 40 nm by an ELISA reader. The increase of the acridine orange green/red ratio was calculated to indicate the dysfunction of lysosomes.

### 4.8. Intracellular Superoxide

The intracellular superoxide was estimated by using a dihydroethidium probe (DHE; Beyotime, Nantong, China), as previously described, with modifications [43]. DHE is cell permeable and could be oxidized to ethidium to emit red fluorescence upon the reaction with intracellular superoxide, thus acting as a specific probe for superoxide [44]. For the assay, after 3 h of exposure to various concentrations of ZnO NPs with the presence of BSA or LNA, HepG2 cells on a black 96-well plate were rinsed once with Hanks’ solution, incubated with 10 µg/mL DHE for 30 min in the dark, and then rinsed with Hanks’ solution. The red fluorescence was read at *E*_x_ 530 ± 25 nm and *E*_m_ 590 ± 35 nm by an ELISA reader.

### 4.9. Intracellular Zn Ions

The accumulation of intracellular Zn ions in HepG2 cells after 3 h of exposure to various concentrations of ZnO NPs with the presence of BSA or 200 µM LNA was measured by using a fluorescent probe Zinquin ethyl ester (Sigma-Aldrich, Saint Louis, MO, USA), as we previously described [13].

### 4.10. ELISA

The supernatants collected from the exposed cells were stored at −20 °C within one month before analysis. The concentrations of interleukin-1β (IL-1β) and IL-6 were measured by using ELISA kits according to the manufacturer’s instructions (Neobioscience Technology Co., Ltd., Guangzhou, China). These commercial kits were based on the double antibody sandwich method, and the concentrations of unknown cytokines were calculated according to the standard curve ranging from 7.8 to 500 pg/mL. The detection limit for both IL-1β and IL-6 is 3.9 pg/mL. We measured cytokines in complete cell culture media containing BSA because it has been suggested that serum proteins could reduce the binding of NPs to cytokines [45].

### 4.11. Statistics

All the data were expressed as means ± standard error (S.E.) of means of 3–5 independent experiments. Two-way ANOVA, followed by a Tukey HSD test, was conducted using R 3.2.2. The *p* value < 0.05 was considered as statistically significant.

## 5. Conclusions

In conclusion, the results from the present study showed that the presence of BSA or BSA-complexed LNA could alter the hydrodynamic sizes, zeta potential, UV-Vis, fluorescence, and synchronous fluorescence spectra of ZnO NPs, which indicated a coating effect of BSA or LNA on NPs. However, the interactions between LNA and ZnO NPs did not significantly affect ZnO NP and BSA induced cytotoxicity, production of intracellular superoxide, accumulation of Zn ions, and the release of inflammatory cytokines in HepG2 cells.
